# Fetal hemoglobin level and nutritional status in patients with sickle cell disease

**DOI:** 10.1186/s12937-016-0181-x

**Published:** 2016-07-08

**Authors:** Sherif M. Badawy

**Affiliations:** 1 Department of Pediatrics, Division of Hematology, Oncology and Stem Cell Transplantation, Ann & Robert H. Lurie Children’s Hospital of Chicago, Feinberg School of Medicine at Northwestern University, 225 E Chicago Ave., Box # 30, Chicago, IL 60611 USA; 2 Department of Pediatrics, Division of Hematology/Oncology, Faculty of Medicine, Zagazig University, Zagazig, Egypt

**Keywords:** Sickle cell disease, Fetal hemoglobin, Hydroxyurea, Nutrition

## Abstract

Hydroxyurea is the only medication approved by the U.S. Food and Drug Administration for sickle cell disease, and there is strong evidence to support the efficacy and the cost effectiveness of using hydroxyurea is patients with sickle cell disease by increasing fetal hemoglobin levels. It is important to clarify the relationship between patients’ nutritional status/intake and fetal hemoglobin levels. In particular, hydroxyurea has been recommended for patients with poor growth, and the recent guidelines from the National Institute of Health suggested offering hydroxyurea to patients as young as nine month old of age.

## Correspondance/Findings

Factors determining sickle cell disease severity, and the relationship between fetal hemoglobin level and patients nutritional status.

## To the editor

With great interest, I have read an article by Mandese et al [1], recently published in Nutrition Journal. The study reported on the nutritional status among children with sickle cell disease (SCD) in relation to disease severity and other morbidity outcomes. The authors reported a negative correlation between fetal hemoglobin levels and the intake of carbohydrates, lipids, iron, phosphorus, vitamins B1, and B2, suggesting a beneficial effect that low intake of these nutritional elements [1]. However, I would like to highlight two points. First, the authors used a number of measures to examine disease severity, however the evidence to support the rationale for choosing the measures was unclear. Second, Mandese et al presented some data in Table 2 in their article that do not match the text [1]. All reported correlations (r^2^) related to intake of different nutritional elements have positive significant values suggesting direct relationship, including mean fetal hemoglobin level, the number of hospitalizations and the number of days for hospitalizations in the year prior. I wonder if the authors could explain this further. Hydroxyurea is the only medication approved by the U.S. Food and Drug Administration for SCD, and there is strong evidence to support the efficacy and the cost effectiveness of using hydroxyurea is patients with SCD by increasing fetal hemoglobin levels [2–4]. Therefore, it is important to clarify the relationship between patients’ nutritional status/intake and fetal hemoglobin levels. In particular, hydroxyurea has been recommended for patients with poor growth [3], and the recent guidelines from the National Institute of Health suggested offering hydroxyurea to patients as young as nine month old of age [5].

**Response from the Authors of Effects of nutritional intake on disease severity in children with sickle cell disease.** *Nutr J* 2016, **15:**46.

Valentina Mandese^1^, Francesca Marotti^2^, Luca Bedetti^1^, Elena Bigi^2^, Giovanni Palazzi^3^ and Lorenzo Iughetti^1,2,3^

^1^Post Graduate School of Pediatrics, ^2^ Pediatric Unit, ^3^Pediatric Oncoematology Unit, Department of Medical and Surgical Sciences for Mothers, Children and Adults, University of Modena and Reggio Emilia, Modena 41124, Italy.

We would like to thank Doctor Badawy for the interesting remarks about our study.

In relation to the first point we think that the number of hospitalizations for vaso-occlusive crisis (VOC), days of hospitalization per year and number of Acute Chest Syndrome (ACS) during life are the best modality to identify patients with worst outcomes. On the other hand, VOC and ACS represent the most frequent complications in sickle cell disease [6–8].

The number of severe episodes of VOC (requiring hospitalization) and of ACS drives the clinicians in the decision to start the therapy with hydroxyurea (HU) or continue the close follow-up of these patients [9]. We reported an inverse correlation between number and day of hospitalizations per year and fetal hemoglobin levels with intake of carbohydrates, lipids, iron, phosphorus, vitamins B1, and B2, but with these data we don’t want to suggest a beneficial effect of low intake of these nutritional elements for these patients. We would like to highlight that these data were unexpected even for us and further studies with more patients are needed to clarify the relationship between nutritional intake and fetal hemoglobin irrespectively from HU therapy. We are aware that HU is the only medication approved by the U.S. Food and Drug Administration and EMA for SCD and that there is a strong evidence to support the efficacy and the cost effectiveness of using HU in patients with SCD even from the first years of life [10–12]. Accordingly, many patients in follow-up in our center are treated with HU for its beneficial effects on SCD.

## Abbreviation

SCD, sickle cell disease
